# Supplementary material for: Machine learning differentiates enzymatic and non-enzymatic metals in proteins
Source: Nat Commun. 2021 Jun 17;12:3712. doi: 10.1038/s41467-021-24070-3 (PMC8211803; doi:10.1038/s41467-021-24070-3)
Supplement: Supplementary file 3 — Description of Additional Supplementary Files [file 41467_2021_24070_MOESM3_ESM.pdf]

Description of Additional Supplementary Files

Supplementary Data 1:

a) Manually examined sites: From the final dataset, 50 sites labelled enzymatic and 50 sites labelled non-enzymatic were randomly sampled for manual inspection using the PDB publication, UniProt, and PyMOL to check if they were metal ions at a catalytic site or non-catalytic site.
b) Feature details: A complete list of features used for machine learning, the category they belong to, and their calculated similarity.
c) Feature set details: All 67 feature set names and the features included in them.
d) Incorrect predictions: The test-set sites that were initially incorrectly predicted and our finding from manual inspection.
e) Feature importance: The relative importance output by our extra-trees MAHOMES model
f) Manually annotated M-CSA: The downloaded M-CSA data with our additional manual annotations. Also includes information for which enzymes and their homologs were removed during the enzymatic labelling process.

Supplementary Data 2:

Final list of sites that were used during the ML process. *SITE_ID* is that site index. *PDB ID* is the crystal structure containing the site. *Chain ID* is the polypeptide identifier for the chain that the site was bound to. *resName1, resName2, resName3,* and *resName4* are the three letter residue codes for the metal ions included in the site, left blank depending on the number of atoms in the site. *seqNum1, seqNum2, seqNum3,* and *seqNum4* are the sequence index in the PDB file for the metal ions included in the site. *Enzyme* is a true for sites identified by our pipeline to be enzymatic and false for those identified as non-enzymatic (the test-set sites found to be mislabeled are not corrected in this file). *Set* is ‘data’ for sites in the dataset and ‘test’ for sites in the test-set. *Resolution (Å)* is the crystal structures resolution. *PDB Dep. Date* is the date the structure was deposited in the PDB. *PDB Classification* and *PDB Macromolecular Name* are from the authors of the crystal structure. *EC No* is the downloaded EC number from the RCSB at the time of data collection. *Uniprot Acc* is the accession code(s) in the UniProt KB database for the polypeptide sequence. *Distance Site moved during relax (Å)* is the distance between the site’s average location in the original and relaxed structure. *Homolog M-CSA ID* is the entry in the M-CSA database for the sequence homolog with the highest E-value, *M-CSA e_val*, which was further used for enzymatic identification. *Homolog M-CSA aligned TM_len, Homolog M-CSA aligned TM_rmsd, Homolog M-CSA aligned TMscore, M-CSA TM_seqID,* and *Homolog M-CSA aligned catalytic residue distance from site* are the results from aligning the site and its structure with the homolog for the corresponding M-CSA entry.

Supplementary Data 3:

The sequence test-set. Each entry starts with a ‘>’ followed by an identifier, which includes uniport accession numbers and one of the SITE_IDs bound to that sequence. The following Boolean value marks weather it is an enzymatic sequence or not. The next line starts the sequence in FASTA format.
